# Supplementary material for: Smart joints: auto-cleaning mechanism in the legs of beetles
Source: Commun Biol. 2022 Sep 28;5:1030. doi: 10.1038/s42003-022-03924-6 (PMC9519561; doi:10.1038/s42003-022-03924-6)
Supplement: Supplementary file 2 — Supplement Information [file 42003_2022_3924_MOESM2_ESM.pdf]

Supplementary information

To the paper: Nadein, K. & Gorb, S. 2022. Smart joints: auto-cleaning mechanism in the legs of beetles. *Communications Biology*.

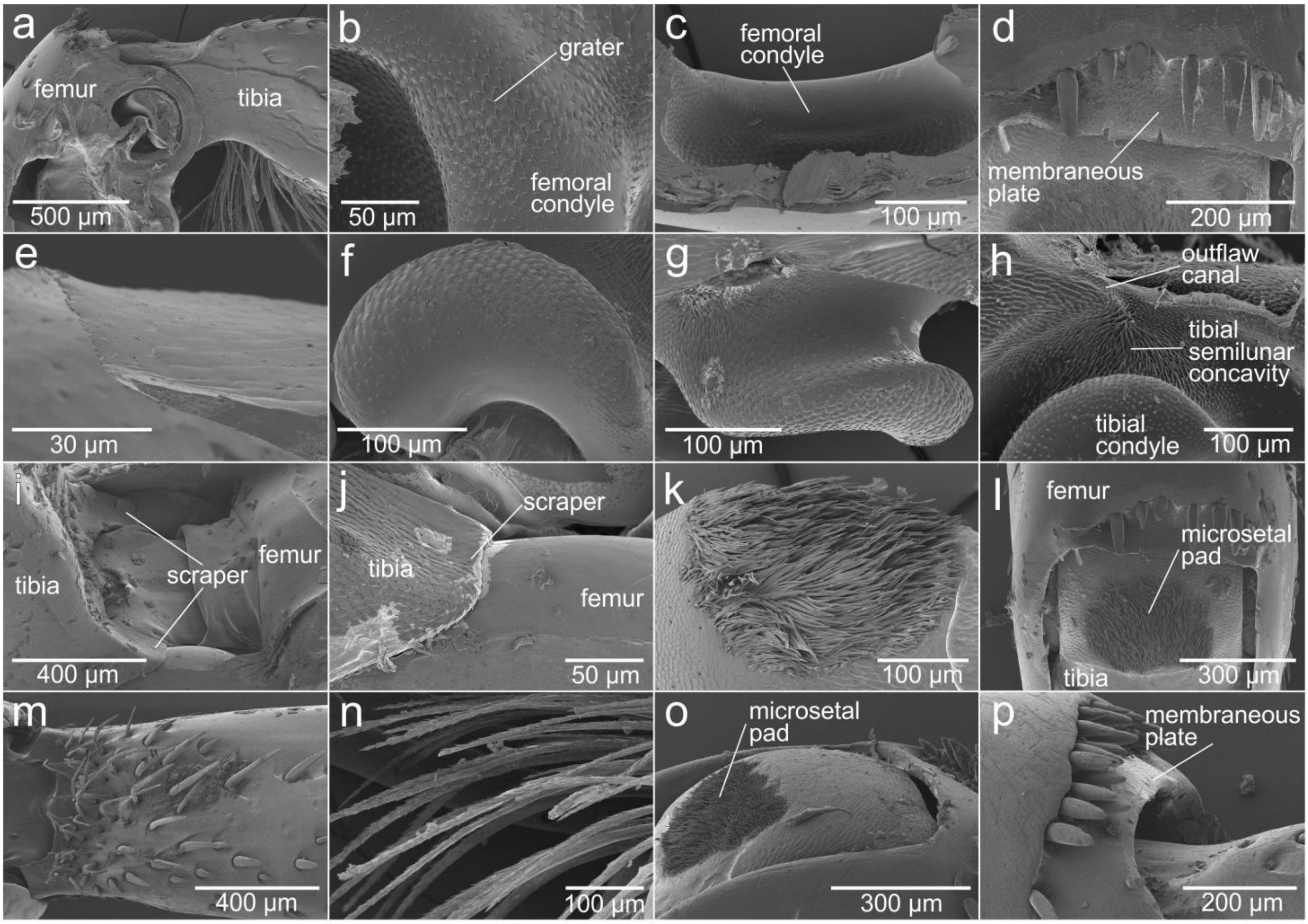

Fig. S1. *Pachnoda marginata*, structure of the femoro-tibial joint, SEM micrographs. a, femoro-tibial joint, parasagittal section, lateral femoral wall is removed; b, grater; c, internal surface of the femoral condyle; d, membraneous plate, dorsal view; e, membraneous plate edge, lateral view; f, tibial condyle, dorsal view; g, tibial condyle, surface contacting femoral condyle; h, surface of the tibial semilunar concavity; i, ventral surface of the femoro-tibial joint; j, scraper, dorso-lateral view; k, microsetal pad, anterolateral view; l, microsetal pad, dorsal view; n, hairy brush, ventral view; m, hairs of hairy brush; o, femoro-tibial joint with flexed tibia, dorsal surface, anterolateral view; p, femoro-tibial joint with extended tibia, dorsal surface, anterolateral view.

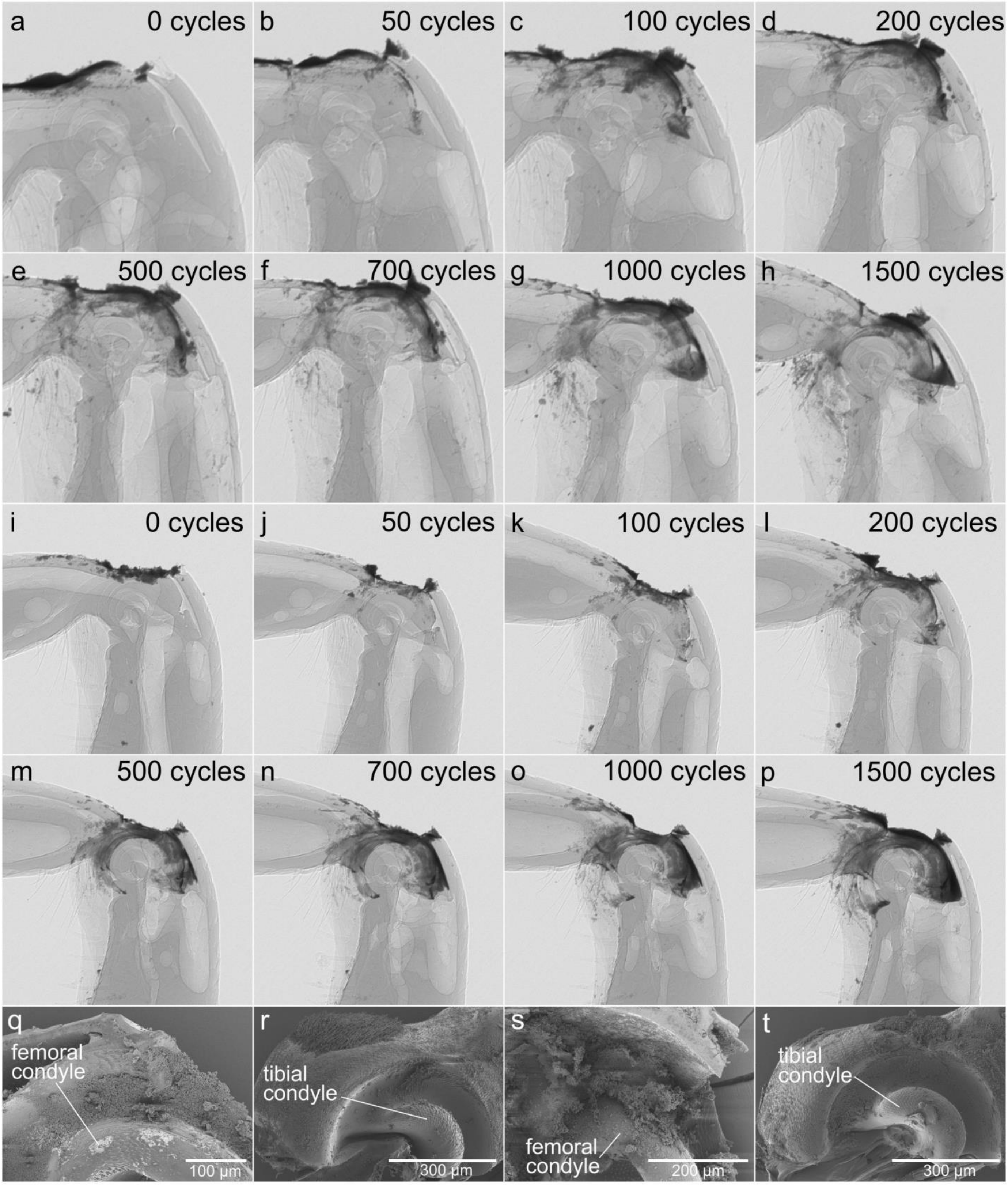

Fig. S2. Experimental results, *Pachnoda marginata*. Contamination of microsetal pad with 1-3 µm metallic particles; a-p, micro-CT; q-t, SEM micrographs; a-h, native state; i-p, microsetal pad and membraneous plate removed. q, femur, sagittal section, native state; r, tibia, lateral view, native state; s, femur, sagittal section, microsetal pad and membraneous plate removed; t, tibia, lateral view, microsetal pad and membraneous plate removed.

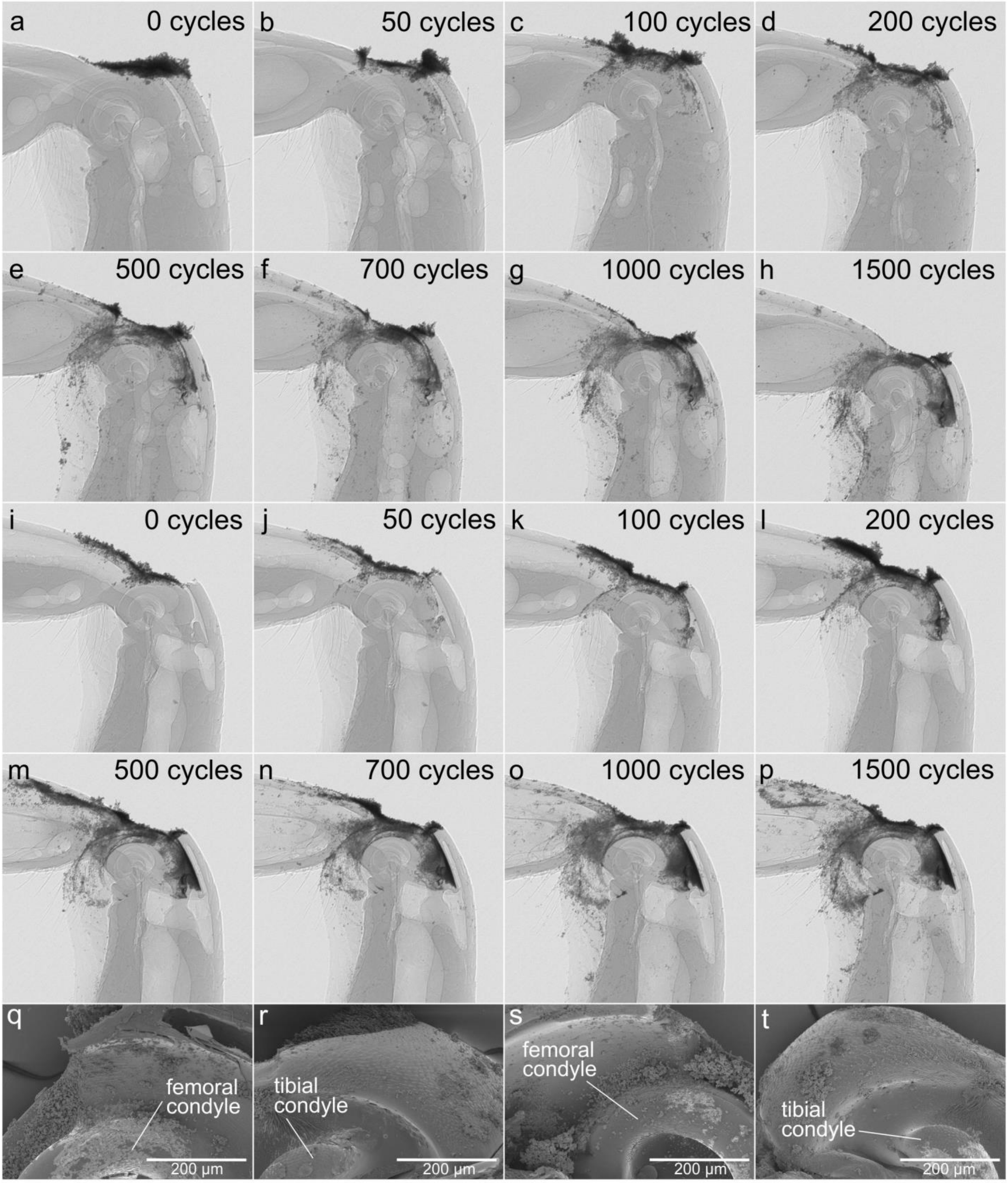

Fig. S3. Experimental results, *Pachnoda marginata*. Contamination of microsetal pad with 2-5 µm metallic particles; a-p, micro-CT; q-t, SEM micrographs; a-h, native state; i-p, microsetal pad and membraneous plate removed. q, femur, sagittal section, native state; r, tibia, lateral view, native state; s, femur, sagittal section, microsetal pad and membraneous plate removed; t, tibia, lateral view, microsetal pad and membraneous plate removed.

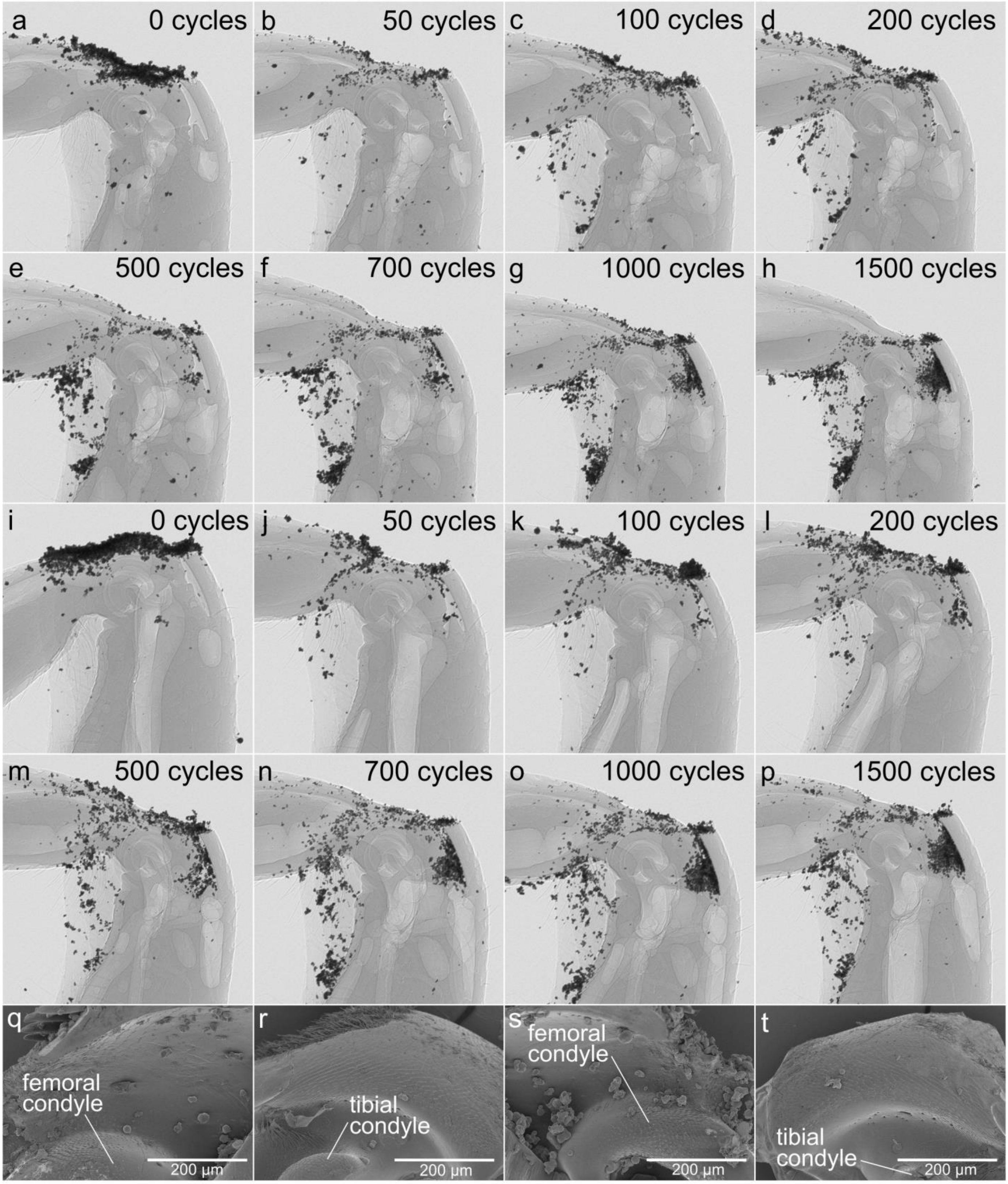

Fig. S4. Experimental results, *Pachnoda marginata*. Contamination of microsetal pad with 20-50 µm metallic particles; a-p, micro-CT; q-t, SEM micrographs; a-h, native state; i-p, microsetal pad and membraneous plate removed. q, femur, sagittal section, native state; r, tibia, lateral view, native state; s, femur, sagittal section, microsetal pad and membraneous plate removed; t, tibia, lateral view, microsetal pad and membraneous plate removed.

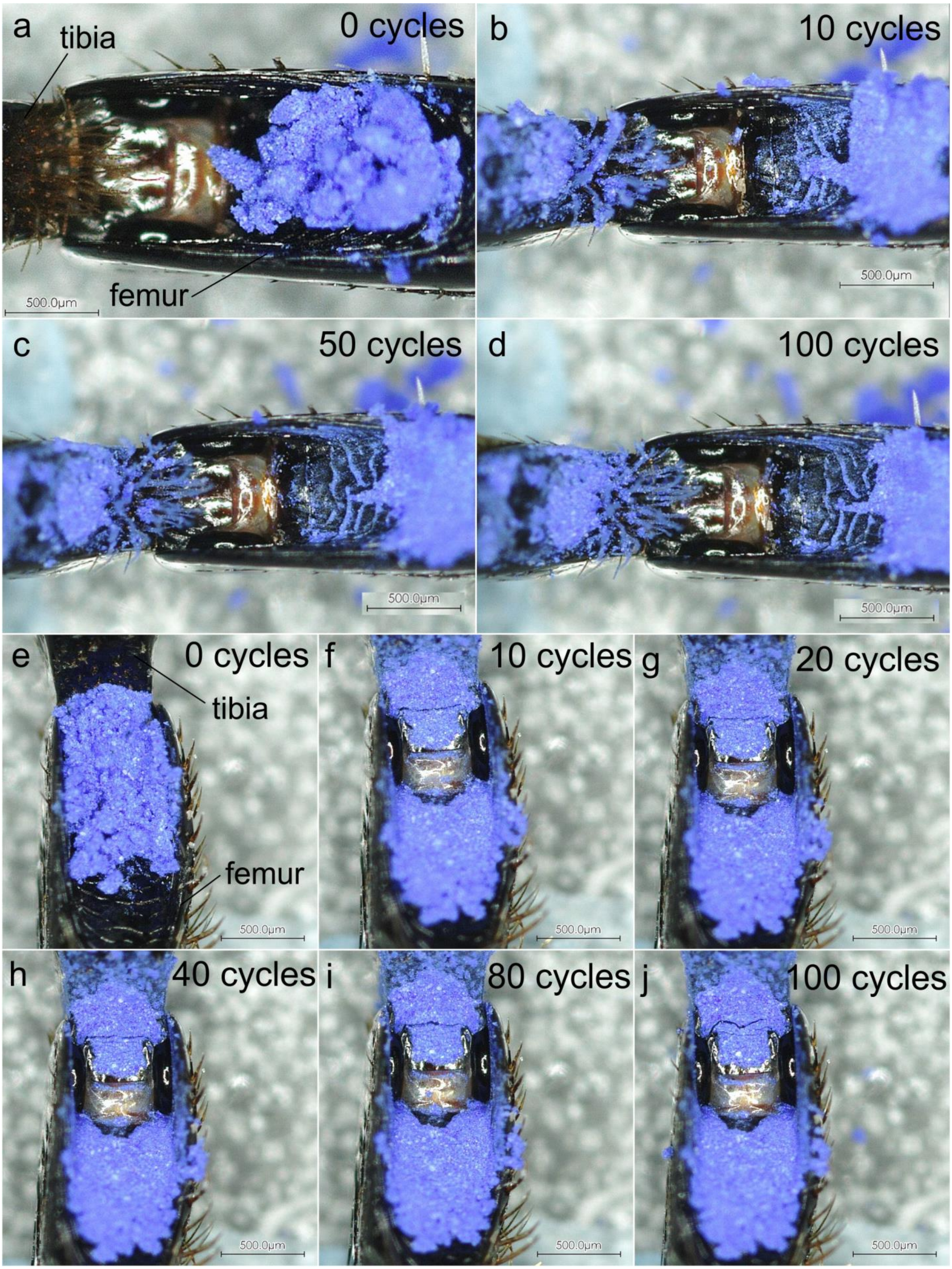

Fig. S5. Experimental results, *Pachnoda marginata*. Contamination of the ventral side of the femoro-tibial joint by iron oxide powder (blue coloured). a-d, experiment with the hairy pad; e-j, experiment with the scraper. Scale bar: a-j, 500 µm.

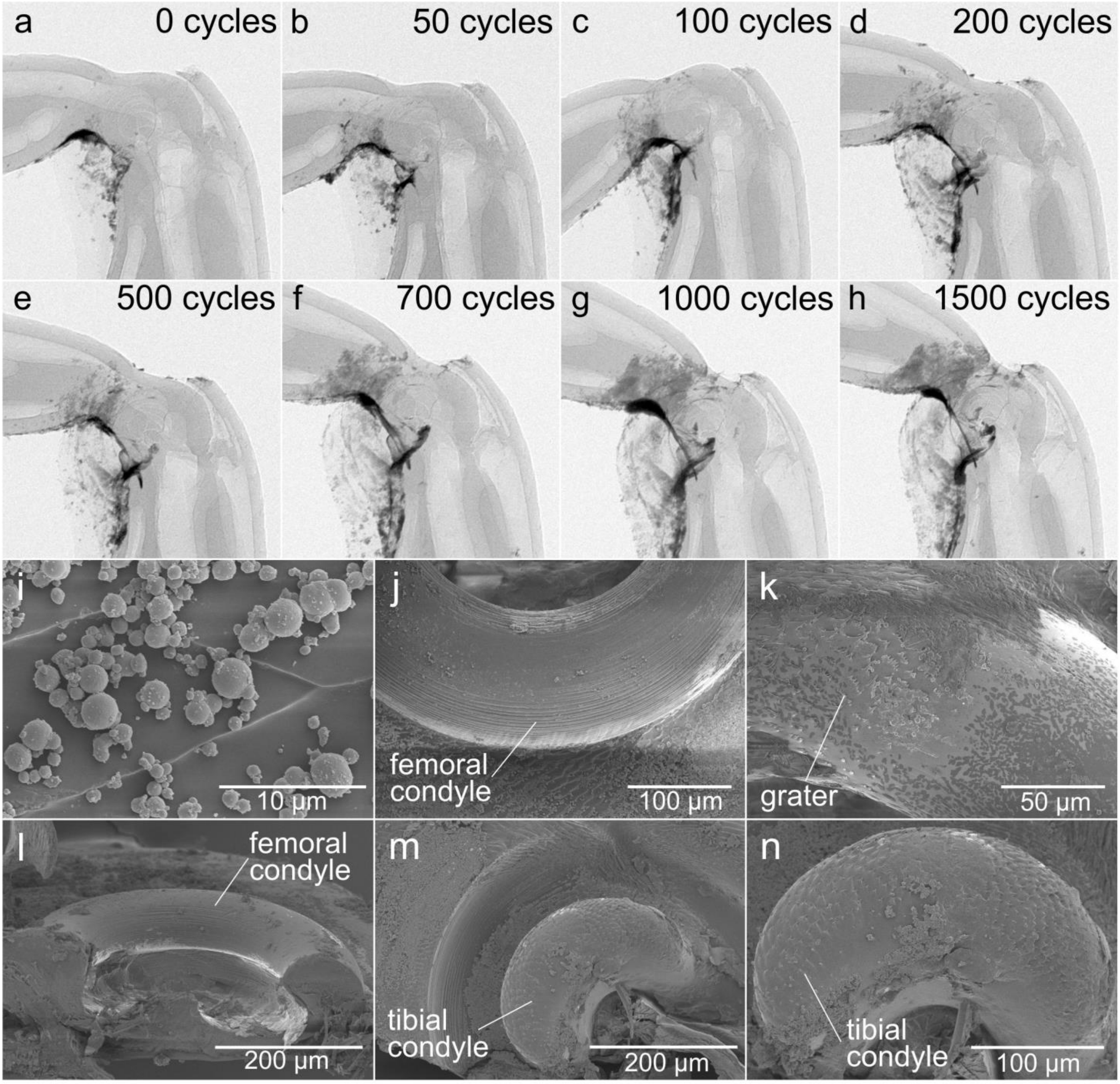

Fig. S6. Experimental results, *Pachnoda marginata*. Contamination of internal joint cavity with 1-3 µm metallic particles; a-h, micro-CT, i-n; SEM micrographs; i, iron particles; j, femoral condyle; k, grater on the femoral condyle; l, internal surface of the femoral condyle; m, tibial counterpart of the femoro-tibial joint; n, tibial condyle.

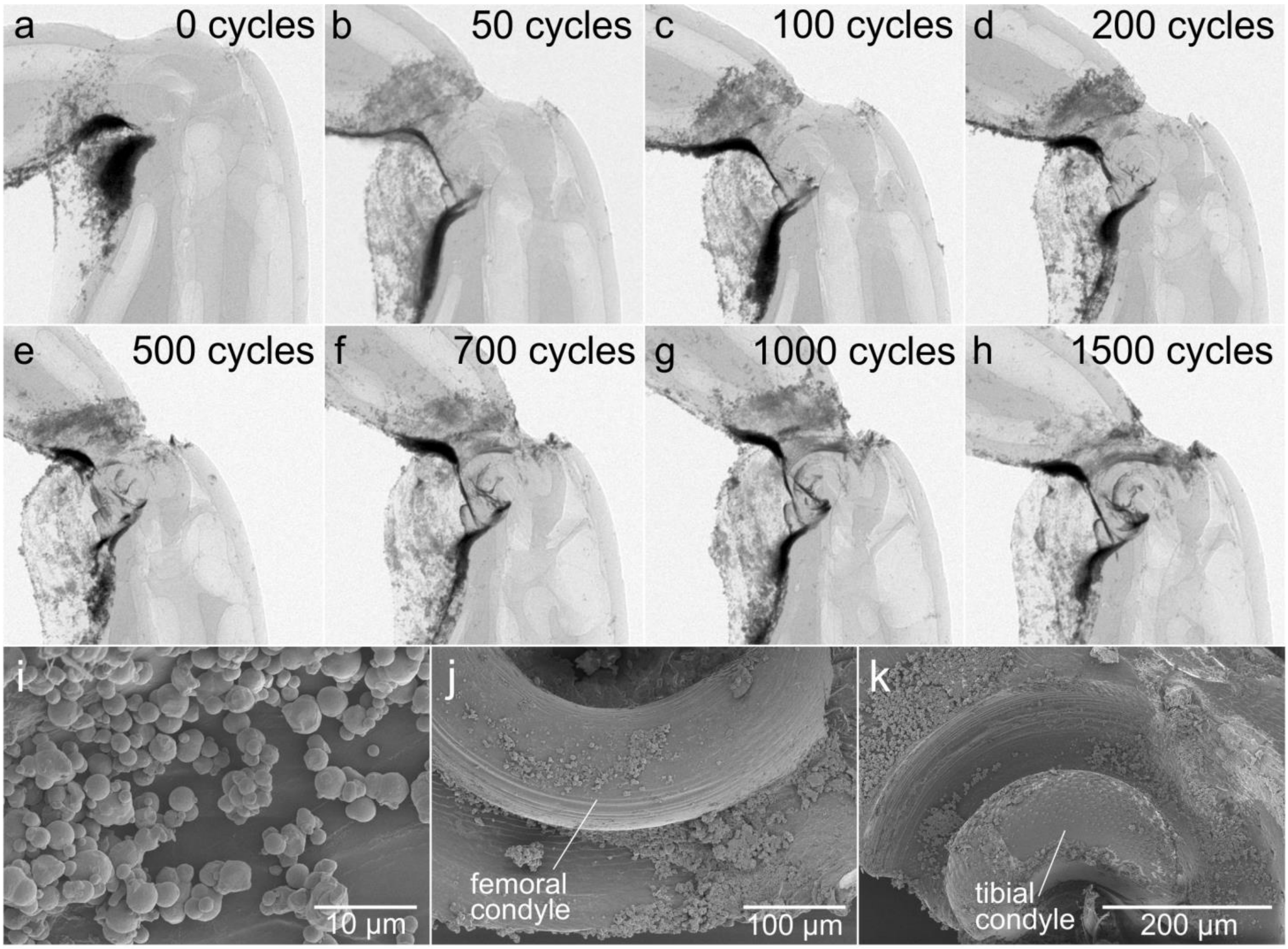

Fig. S7. Experimental results, *Pachnoda marginata*. Contamination of internal joint cavity with 2-5 µm metallic particles; a-h, micro-CT; i-k, SEM micrographs; i, iron particles; j, femoral condyle; k, tibial counterpart of the femoro-tibial joint.

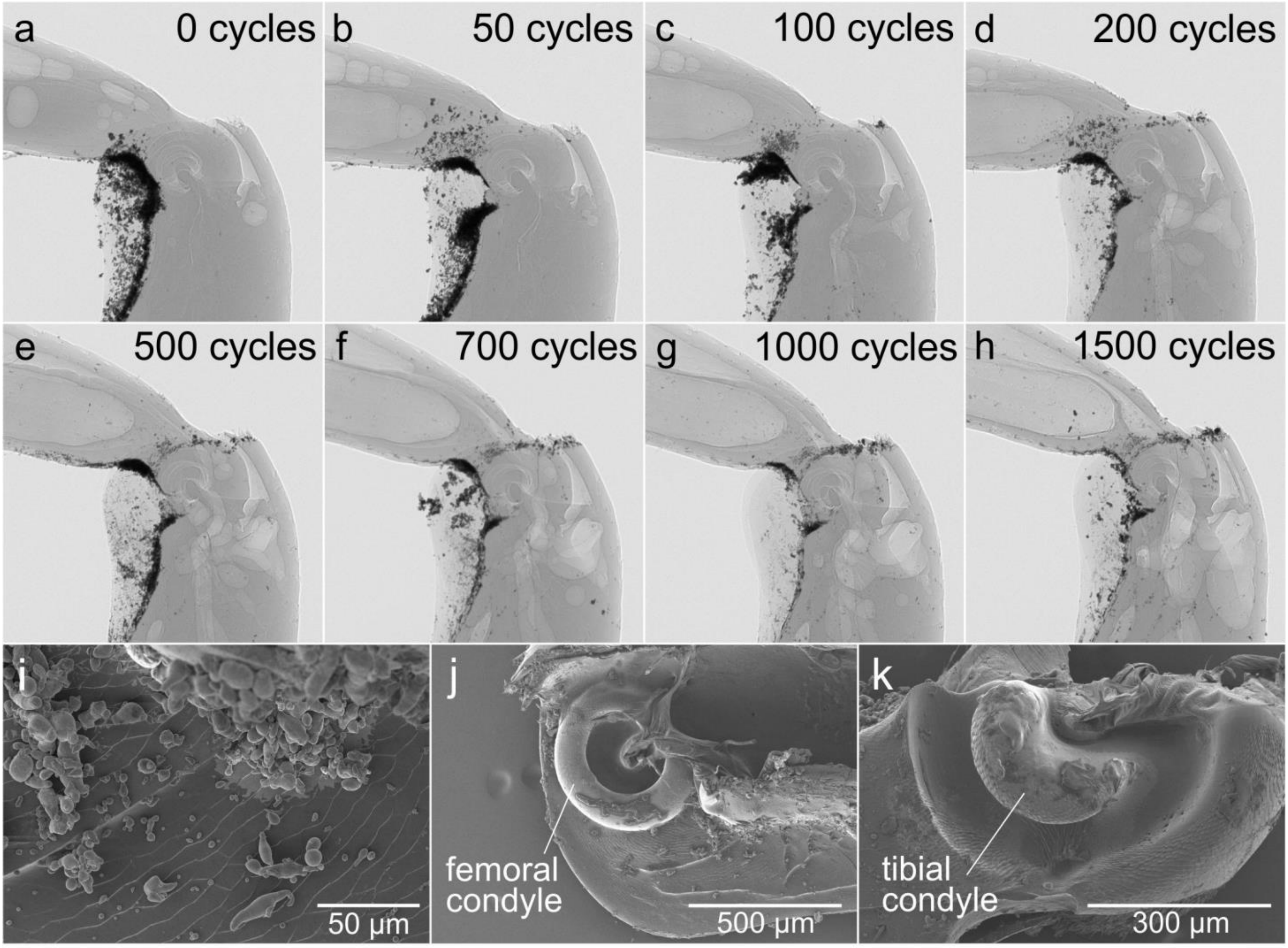

Fig. S8. Experimental results, *Pachnoda marginata*. Contamination of internal joint cavity with 10-30 µm metallic particles; a-h, micro-CT; i-k, SEM micrographs; i, iron particles; j, femoral counterpart of the femoro-tibial joint, sagittal section; k, tibial counterpart of the femoro-tibial joint.

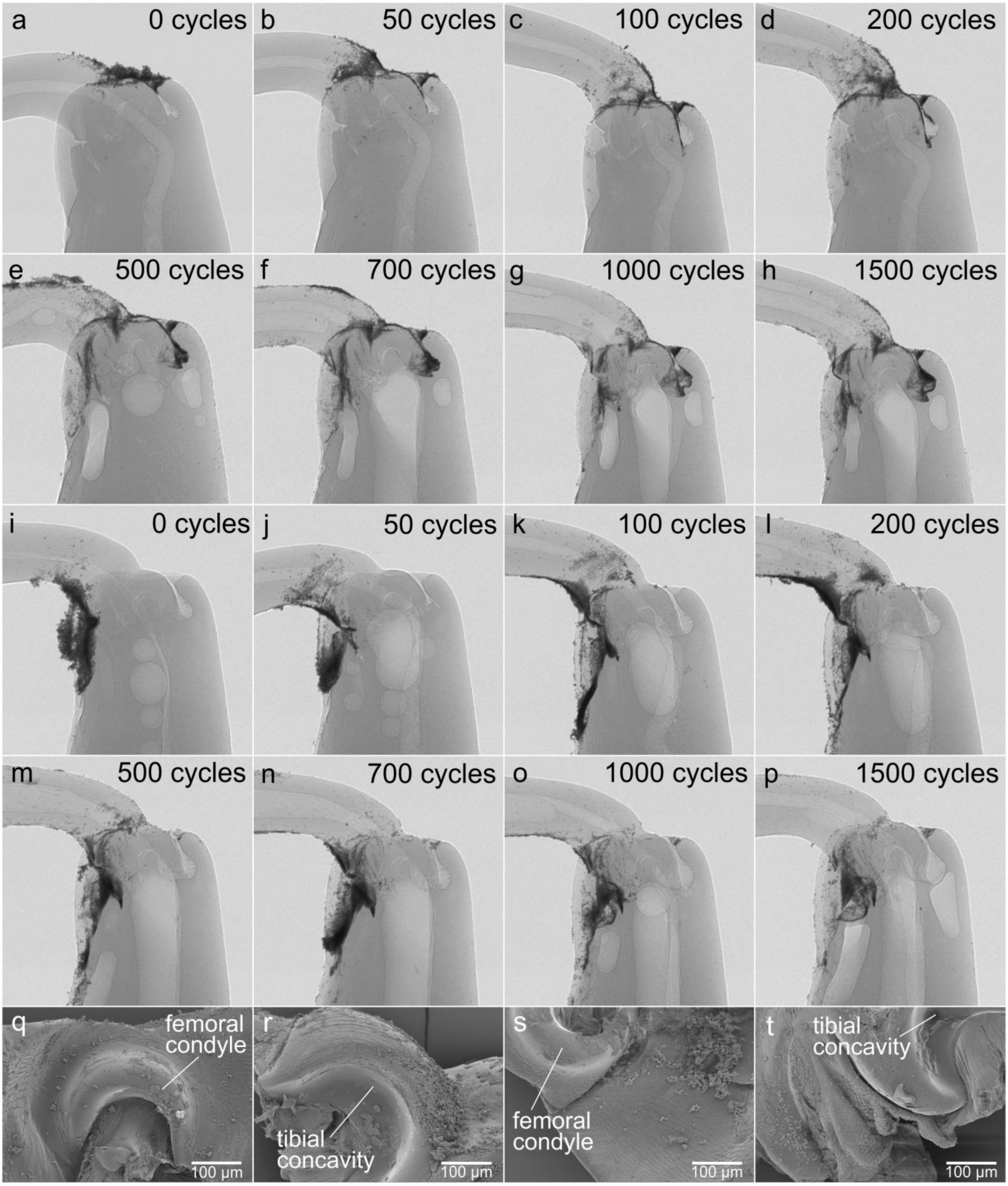

Fig. S9. Experimental results, *Zophobas morio*. Contamination of joint cavity with 2-5 µm metallic particles; a-h, q, r, contamination through the dorsal gap; i-p, s, t, contamination through the ventral gap; a-p, micro-CT; q-t, SEM micrographs; q, femur, sagittal section; r, tibia, lateral view, s, femur, sagittal section, t, tibia, lateral view.

Table S1. Results of testing the effectiveness of microsetal pad/membraneous plate cleaning by contamination with metal particles of different sizes (1-3 µm, 2-5 µm, 20-50 µm). The data are presented as the projected areas (mm^2) occupied by the particles (see section Material and methods).

Cycles	Erased, area (mm^2)			Native, area (mm^2)		
	1-3 µm	2-5 µm	20-50 µm	1-3 µm	2-5 µm	20-50 µm
50	0.064	0.063	0.044	0.036	0.060	0.031
100	0.097	0.103	0.073	0.087	0.086	0.044
200	0.127	0.166	0.080	0.090	0.121	0.049
500	0.248	0.190	0.087	0.102	0.153	0.054
700	0.285	0.206	0.091	0.125	0.178	0.061
1000	0.292	0.233	0.100	0.163	0.200	0.082
1500	0.368	0.259	0.108	0.207	0.215	0.090
